# Neuroimmune System as a Driving Force for Plasticity Following CNS Injury

**DOI:** 10.3389/fncel.2020.00187

**Published:** 2020-07-23

**Authors:** Micaela L. O’Reilly, Veronica J. Tom

**Affiliations:** Department of Neurobiology and Anatomy, Marion Murray Spinal Cord Research Center, Drexel University College of Medicine, Philadelphia, PA, United States

**Keywords:** neuroinflammation, plasticity, neurotrauma, injury, stroke, cytokines, growth factors, sprouting

## Abstract

Following an injury to the central nervous system (CNS), spontaneous plasticity is observed throughout the neuraxis and affects multiple key circuits. Much of this spontaneous plasticity can elicit beneficial *and* deleterious functional outcomes, depending on the context of plasticity and circuit affected. Injury-induced activation of the neuroimmune system has been proposed to be a major factor in driving this plasticity, as neuroimmune and inflammatory factors have been shown to influence cellular, synaptic, structural, and anatomical plasticity. Here, we will review the mechanisms through which the neuroimmune system mediates plasticity after CNS injury. Understanding the role of specific neuroimmune factors in driving adaptive and maladaptive plasticity may offer valuable therapeutic insight into how to promote adaptive plasticity and/or diminish maladaptive plasticity, respectively.

## Introduction

Damage to the central nervous system (CNS) in the form of traumatic brain injury (TBI), spinal cord injury (SCI), or stroke are among the leading causes of disability, morbidity, and mortality (Rubiano et al., 2015). Depending on the location and severity of the primary insult, individuals affected by CNS injury often exhibit early impairments in motor, sensory, and autonomic functions which greatly impair their quality of life and overall health. Over the days, weeks, months, and years which proceed the initial trauma, extensive plasticity throughout the nervous system contributes to the regaining of functional activity in numerous neural circuits. The mechanisms underlying this plasticity has been an important topic of research within the field, as therapeutic targeting of these circuits may promote the recovery of crucial functions such as locomotion, sensation, and bladder/bowel function. Moreover, understanding how this plasticity contributes to the development of detrimental consequences, such as chronic pain, spasticity, and autonomic dysreflexia, will provide a foundational basis for targeting maladaptive vs. adaptive plasticity.

The neuroimmune system is a major driving factor in plasticity throughout the neuraxis. Immediate activation of both central and peripheral neuroimmune and inflammatory processes contributes to both reparative and pathological activity that can persist in a chronic state of inflammatory cascades (Jassam et al., 2017; Chen et al., 2018). Moreover, this injury-induced neuroimmune activity, directly and indirectly, influences cellular, anatomical, and physiological plasticity that can be ultimately beneficial or detrimental, depending on the context. Due to these juxtaposing effects, neuroinflammation is often regarded as a controversial target for therapeutic modification. However, understanding the diverse and intricate mechanisms by which the neuroimmune system mediates plasticity at a local and global level will provide crucial insight as to how neural circuits are differentially altered and ultimately result in beneficial and/or deleterious function. Moreover, this insight will be of great clinical and scientific importance. Therefore, the goal of this review is to provide an overview of how the neuroimmune system shapes plasticity following CNS injury and how neuroimmune-mediated plasticity drives functional and/or dysfunctional outcomes. Additionally, we will discuss advancements in pre-clinical and clinical therapies that target neuroimmune and inflammatory activity to enhance or suppress factors associated with adaptive or maladaptive outcomes, respectively.

## Early Inflammogenesis and Vascular Plasticity

Primary insult to the CNS causes immediate damage to the blood-brain barrier (BBB), blood-spinal barrier (BSB), and the blood-cerebrospinal fluid barrier (BCSF), as well as to neuro-axonal structures and tissue deformation. Consequently, this homeostatic disruption triggers a wave of inflammatory cascades *via* activation of innate residential (microglia and astrocytes), peripheral (neutrophils and monocytes/macrophages), and adaptive (T- and B-lymphocytes) immune cells, ultimately contributing to mechanisms of secondary injury that may persist for months or years (Wang et al., 2007; Donnelly and Popovich, 2008; Anwar et al., 2016; Jassam et al., 2017). Although astrocytes are not typically classified as a neuroimmune cell, the ability of these cells to produce and secrete numerous immune factors is a distinguishing characteristic of immunocompetent cells (Dong and Benveniste, 2001; Farina et al., 2007; Brambilla, 2019) and warrants including astrocytes in this category. Thus, for this review, astrocytes will be considered as a glial immune cell. Until recently, when the transmembrane surface protein Tmem119 was discovered as a specific marker for microglia (Bennett et al., 2016; Kaiser and Feng, 2019), it was extremely difficult to distinguish peripherally-derived macrophages from microglia in CNS tissue. Thus, the vast majority of literature on this topic utilizes insufficient markers to distinguish microglia from macrophages. For these reasons, these cells will be grouped as microglia/macrophages unless identified singularly.

Throughout the acute post-injury phase, neuroimmune and inflammatory cells are crucial components involved in driving reparative processes. Upon detecting cues of cellular and tissue damage [e.g., inflammatory chemokines (CCL2, CXCL1, CXCL2, CCL21), ATP, glutamate, heat shock proteins (HSPs), neuregulin-1 (NRG1), high mobility group box 1 protein (HMGB1), fibronectin, etc., Calvo et al., 2010; Grace et al., 2014; Jassam et al., 2017], resting microglia are immediately activated and initiate the release of proinflammatory amplifiers such as interleukin (IL)-1β and IL-18 (Olson and Miller, 2004). Coupled with endogenous alarmins, antigens, and inflammatory signals, this microglial response further stimulates the infiltration of neutrophils, monocytes/macrophages, lymphocytes, and dendritic cells to the injury site (Donnelly and Popovich, 2008). These temporal cascades are further correlated with increased expression of inflammatory mediators [e.g., tumor necrosis factor-alpha (TNFα), IL-1β, IL-6, reactive oxygen species (ROS), etc.,] and neurotrophic factors [e.g., brain-derived neurotrophic factor (BDNF), glial cell-line derived neurotrophic factor (GDNF), nerve growth factor (NGF), NT-3, etc., Donnelly and Popovich, 2008; Jin et al., 2010; da Silva Meirelles et al., 2017], which contribute to driving cellular, axonal, and anatomical plasticity described below in more detail.

These immune cells, as well as the factors that they produce, directly and indirectly, modify key components involved in vascular function and contribute to a secondary wave of increased vascular permeability (Donnelly and Popovich, 2008; Sprague and Khalil, 2009). Specifically, sites of enhanced vascular permeability are spatially correlated with clusters of activated microglia (Popovich et al., 1996) and injury-induced expression of matrix metalloproteinase-9 (MMP-9) is implicated as a potent regulator of microglial activation and macrophage infiltration by increasing vascular permeability (Hansen et al., 2013, 2016). This increased permeability and enhanced infiltration of immune cells are furthered by the release of pro-inflammatory cytokines, such as TNFα and IL-1β which are immediately and persistently upregulated after injury and can further enhance vascular permeability (Schnell et al., 1999; Donnelly and Popovich, 2008; Mironets et al., 2018, 2020). Through these mechanisms, the neuroimmune system can drive vascular plasticity by establishing a feed-forward cycle of increased permeability and widespread leukocyte infiltration and inflammation throughout the parenchyma. The persistence of this cycle may lead to further long-lasting changes in the BBB, BSB, and/or BCSF and contribute to plasticity distal to the injury site as well as increase infection susceptibility (Haruwaka et al., 2019).

Interestingly, the effects of this vascular plasticity can be beneficial *and* detrimental. Enhanced vascular permeability supports the infiltration of leukocytes, which, in turn, exert crucial roles in containing damage, regulating cellular activity, and supporting neuroprotective processes by interacting with resident neuroimmune cells to further regulate inflammatory cascades. Within the lesion core, immune responders recruited from the periphery (monocyte-derived macrophages, neutrophils) aid residential microglia in phagocytosing debris from necrotic cells, myelin, and damaged tissue (Trivedi et al., 2006; Russo and McGavern, 2015; Jassam et al., 2017). Through this phagocytic activity, macrophages, neutrophils, and microglia produce a slew of harmful factors, including ROS, inflammatory cytokines, and cytotoxins (Liu et al., 2000; Dong and Benveniste, 2001; Trivedi et al., 2006; Donnelly and Popovich, 2008; Wang, 2018).

To protect healthy tissue from this toxicity and limit the expansion of the secondary injury site, a scar comprised of reactive astrocytes, microglia, fibroblasts, and oligodendrocyte precursor cells (OPCs) forms around the lesion core (Yiu and He, 2006; Burda et al., 2016; Hackett and Lee, 2016). Within this region, activated glia secrete extracellular matrix proteins, including chondroitin sulfate proteoglycans (CSPGs) and semaphorins (Silver and Miller, 2004; Fawcett, 2006; Burda et al., 2016; Sims and Yew, 2017), which limit axonal regrowth through the injury site. In spared regions distal to the injury site, microglia/macrophages continue to clear debris, albeit very inefficiently, produced by Wallerian degeneration of axonal tracts, oligodendrocyte apoptosis, and chronic, secondary demyelination of axons that are surrounded by astrocytic processes (David and Lacroix, 2003; Buss et al., 2004; Totoiu and Keirstead, 2005; Vargas and Barres, 2007). Throughout each of these acute post-injury cellular processes, cross-talk between residential and infiltrating immune cells, as well as between innate and adaptive immune cells, forms a crucial feedback network that further modifies the inflammatory environment and influences tissue pathology post-injury (Bradbury and Burnside, 2019). For example, infiltrating macrophages are crucial for scar formation and fibroblast recruitment (Zhu et al., 2015; Mescher, 2017), and secretion of transforming growth factor-beta (TGF-β1) by microglia/macrophages is suggested to stimulate scar formation following stroke (Doyle et al., 2010).

Taken together, the ability of the neuroimmune system to enhance vascular permeability and increase leukocyte infiltration is highly advantageous early after injury, as residential and circulating immune-responders function together to clear debris, contain damage, minimize secondary injury, and promote tissue remodeling. Importantly, however, chronic, persistent activation and accumulation of immune cells may result in detrimental, non-resolving tissue pathology and scarring. For example, persistent phagocytosis *via* microglia/macrophages and neutrophils contributes to a continued loss of neurons, synaptic inputs, and conduction activity which ultimately leads to substantial cellular and anatomical plasticity, as detailed in the following section. Therefore, although enhanced vascular plasticity supports the infiltration of circulating immune cells to aid residential immune factor-producing cells in mediating tissue pathology, such persistent vascular permeability and subsequent cell infiltration can become detrimental to recovery over time. Through understanding the mechanisms underlying vascular permeability and the time-dependent properties of infiltrating and residential immune cells, these properties may be further optimized for the delivery of therapies that would be otherwise unable to cross the BBB, BSB, and/or BCSF.

The duality of neuroimmune-mediated activity and their functional consequences is exemplified by studies examining microglia/macrophage depletion and astrogliosis. The abundance of inflammatory cells and cytotoxic factors that fill the lesion epicenter is crucial for clearing debris and tissue reconstruction, but can also exacerbate tissue damage, lesion pathology, and expand the secondary injury site (Mallat and Chamak, 1994; Gensel et al., 2009). The complexity of the impact of these cells after injury becomes further apparent when they are selectively and conditionally ablated. For example, depletion of hematogenous macrophages *via* liposomal clodronate attenuates secondary injury and improves locomotor function in models of SCI and ischemia (Popovich et al., 1999; Popovich and Hickey, 2001; Zhu et al., 2015; Ma et al., 2016). Contrary to this, other studies have demonstrated that depletion of circulating monocytes and monocyte-derived macrophages *impairs* functional recovery (Shechter et al., 2009), and implantation of macrophages into SCI lesion epicenter and parenchyma promotes functional locomotor recovery (Rapalino et al., 1998). An explanation for such dichotomy may lie in the heterogeneity of microglia/macrophage cell populations, as microglia/macrophages are influenced by the microenvironment to adopt a phenotype along a spectrum of two polarized states: “classically activated” pro-inflammatory M1 cells and “alternatively activated” anti-inflammatory M2 cells that foster repair (Mills et al., 2000; Mantovani et al., 2004; Kigerl et al., 2009; David and Kroner, 2011). Emerging evidence also supports such cellular heterogeneity and phenotypic duality concerning astrocytes (Anderson et al., 2014), as scar-forming reactive astrocytes act as a major barrier to regeneration due to their production of proteoglycans and other growth-inhibitory extracellular matrix molecules (Silver and Miller, 2004); yet reactive astrocytes are also crucial and necessary for limiting tissue degeneration, cell infiltration, and improving functional motor activity (Bush et al., 1999; Faulkner et al., 2004; Myer et al., 2006). In a detailed review by Bradbury and Burnside (2019) that goes beyond the scope of the present review, the neuroimmune system exhibits a complex and diverse role in mediating neural plasticity after CNS injury that can be portrayed as “good vs. bad”; however, the multifaceted properties of neuroimmune and inflammatory cells are an even further impetus to understand targeting *specific* components of this system that drive *specific* plasticity to enhance therapeutic outcomes.

## Neuroimmune-Mediated Cellular and Synaptic Plasticity Post-Injury

Throughout early inflammogenesis and secondary injury, there is an initial depression of cellular excitability local and remote to the injury site (D’Amico et al., 2014; Carron et al., 2016). Within the context of SCI, this often manifests as spinal shock by exhibiting symptoms of hyporeflexia, hypotonicity, depression of sympathetic reflexes, and loss of sensation below the injury (Ditunno et al., 2004). Acute development of this cellular hypoactivation is caused by immediate cell death, tract severing, and overall loss of pre-synaptic inputs induced by the trauma, as well as secondary apoptosis and demyelination caused by glutamate excitotoxicity (Park et al., 2004). Moreover, chronic and progressive axonal degeneration and demyelination results in further loss of inputs, as well as slower axonal signaling, reduced conduction velocity, hyperpolarized resting membrane potentials, and an overall decrease in action potential propagation sufficient for post-synaptic cell polarization (Beirowski et al., 2005; Totoiu and Keirstead, 2005; Gaudet et al., 2011). This initial hypoactivation is observed in both sensory and motor neurons, as well as interneurons following TBI (Johnstone et al., 2014; Carron et al., 2016), SCI (Ditunno et al., 2004; D’Amico et al., 2014), and stroke (Carmichael, 2012). However, in the weeks and months that follow a CNS injury, these affected neural circuits progressively adapt to the loss of inputs and hypoactivity consequent of the neurotrauma. Extensive synaptic and cellular plasticity contribute to this rebound in cellular activity, which results in a similar effect in different types of neurons: enhanced excitability and diminished inhibition. The functional consequences of such changes can be both adaptive and maladaptive, depending on the destination of this cellular output and the context.

Numerous studies have demonstrated that neuroimmune cells and the factors they produce are crucial components involved in driving cellular plasticity. This section will highlight the primary mechanisms by which astrocytes, microglia/macrophages, and immune mediators influence glutamatergic activity, synaptic transmission, and excitatory-to-inhibitory balance to ultimately establish a pro-excitatory forward feedback loop. Furthermore, this section will highlight key factors involved in each of these mechanisms and which may prove to be valuable therapeutic targets for promoting recovery.

### Enhanced Neuronal Excitability

Following CNS injury, neuroimmune and inflammatory factors spread throughout the nervous system and influence cellular activity both local and remote to the injury epicenter. This is observable early after injury, as activated microglia, monocyte-derived macrophages, and astrocytes can, directly and indirectly, regulate glutamatergic excitotoxicity, synaptic transmission, and neuronal activity. Dysregulation of the excitatory neurotransmitter glutamate after an injury is a key component contributing to aberrant neuronal activity and neurotoxicity, ultimately influencing secondary injury and functional outcomes. Normally, cross-talk between astrocytes and microglia/macrophages, as well as other glial cell types, regulate ion and neurotransmitter homeostasis *via* direct synapse formation and/or indirectly *via* cytokine signaling (Sofroniew and Vinters, 2010; Szepesi et al., 2018).

Astrocytes, in particular, are widely considered to be principal regulators of neuronal activity and exert a crucial role in regulating neurotransmitter release and clearing excess glutamate from the extracellular space *via* expression of the glutamatergic transporters excitatory amino acid transporter 1 (EAAT1) and EAAT2 (Schousboe et al., 2013; Gaudet and Fonken, 2018). However, these crucial homeostatic functions of astrocytes are disrupted immediately upon injury. Within hours post-injury, reactive astrocytes exhibit reduced expression of EAAT1 and EAAT2, thereby enhancing excitatory synaptic transmission and excitotoxicity (van Landeghem et al., 2001; Xin et al., 2009; Grace et al., 2014). Reduced expression of EAAT2 specifically in astrocytes is also associated with increased neuronal loss and apoptosis, exacerbated secondary injury, and worsened locomotive function (Lepore et al., 2011). Furthermore, the release of neuroimmune mediators such as TNFα, IL-1β, IFNγ, and ROS impair glutamate metabolism and clearance by astrocytes (Chao et al., 1995; Grace et al., 2014; Haroon et al., 2017).

Microglia and macrophages also influence glutamate homeostasis post-injury, as elevated expression of microglia-derived TNFα elicits excess glutamate release from astrocytes (Bezzi et al., 2001). Moreover, persistent activation of microglia/macrophages, as observed in a chronic injury microenvironment, further contributes to sustained, elevated production of pro-inflammatory cytokines and enhances the release of glutamate (Hanisch, 2002; Domercq et al., 2013; Donat et al., 2017). However, while such studies may suggest that microglia and macrophages are detrimental to neuronal survival and lesion pathology, evidence demonstrates that depletion of these neuroimmune cells results in increased cell loss (Bellver-Landete et al., 2019) and enhanced excitotoxicity (Vinet et al., 2012), suggesting a neuroprotective role for these cells. This is further exemplified by findings demonstrating that microglia/macrophages exhibit *increased* expression of EAAT1 and EAAT2 following injury, and are therefore suggested to exert a compensatory role in regulating extracellular glutamate levels and controlling excitotoxicity (Rimaniol et al., 2000; van Landeghem et al., 2001). Although this compensatory activity does not appear to be sufficient to mitigate the overall increase in glutamate activity to confer significant, robust neuroprotection, this highlights the duality of microglia/macrophages (i.e., being detrimental or beneficial, depending on context) in mediating excitatory activity and that neuroimmune mediators are a crucial component involved in driving such plasticity.

In addition to these intrinsic mechanisms by which astrocytes and microglia/macrophages influence neuronal excitability, these neuroimmune cells drive further plasticity within the post-synaptic cell *via* direct synapses with nearby neurons and glial cells (Miyamoto et al., 2016; Akiyoshi et al., 2018). Although these direct synaptic formations are most often discussed concerning anatomical plasticity (discussed in more detail in the following section), heightened cellular excitability and homeostatic imbalance of glutamate following injury ultimately induces more excitatory postsynaptic currents (EPSCs), elicits increased glutamatergic synapse formation, and enhances excitability throughout the neural circuit (Chung et al., 2015; Akiyoshi et al., 2018).

The secretion of pro-inflammatory cytokines by these neuroimmune cells is among the most predominant mechanisms by which the neuroimmune system drives synaptic plasticity and ultimately influences circuit signaling. As reviewed by Grace et al. (2014), mediators such as TNFα, IL-1β, IL-17, ROS, and chemokines CCL2 and CXCL1 induce post-synaptic sensitization by increasing synaptic transmission (Beattie et al., 2002; Viviani et al., 2003; Stellwagen et al., 2005; Zhang et al., 2013). Numerous studies have demonstrated that TNFα increases surface expression of AMPA receptors (Beattie et al., 2002; Stellwagen et al., 2005) and can indirectly increase NMDA receptor expression *via* extracellular signal-regulated kinases (ERKs; Xu et al., 2010; Grace et al., 2014; Olmos and Lladó, 2014). Additionally, IL-1β, IL-17, and ROS have been shown to enhance post-synaptic NMDA receptor activation by increasing phosphorylation of NMDA receptor subunits NR1 and NR2A/B (Viviani et al., 2003; Gao et al., 2007; Meng et al., 2013). Consistent with this, TNFα and IL-1β enhance AMPA- and NMDA-induced currents (Kawasaki et al., 2008), and TNFα alters neuronal electrophysiological threshold properties to ultimately increase excitatory post-synaptic activity (Spicarova et al., 2011; Olmos and Lladó, 2014; Chen et al., 2015). These cytokines may further drive such cellular plasticity *via* activation of NF-κB, a transcription factor complex that is activated by numerous inflammatory and immune agents. Specifically, activation of NF-κB signaling *via* TNFα application has been shown to increase neuronal expression of glutamatergic receptors and inhibition of NF-κB prevents this effect (Yu et al., 2002). Enhanced activation of NF-κB is also associated with increased excitatory synapses, fewer inhibitory synapses, hyperexcitable neuronal activity, and an overall shift in the balance of excitatory to inhibitory (E/I) activity, favoring increased glutamatergic presynaptic activity, increased spontaneous EPSCs, and increased hyperexcitability (Yu et al., 2002; Shim et al., 2011; Dresselhaus and Meffert, 2019). Importantly, persistent activation of NF-κB following injury also increases transcription of pro-inflammatory factors and IκB family members, thereby establishing a proinflammatory autocrine loop to perpetuate injury-induced inflammation and enhance excitatory cellular activity (Shim et al., 2011; Shih et al., 2015; Mironets et al., 2018).

Together, astrocytes and microglia/macrophages influence cellular excitability following injury directly *via* central synaptic and cytokine signaling as well as indirectly *via* crosstalk signaling between neuroimmune cells. The persistent upregulation of numerous neuroimmune mediators for weeks, months, and even years post-injury ultimately results in sustained biochemical, electrophysiological, and synaptic changes. Over time, this long-term plasticity drives the formation of pro-excitatory feedback loops, which perpetuate heightened cellular excitability and excitotoxicity (Donnelly and Popovich, 2008; Yawata et al., 2008). This is exemplified by motoneurons following chronic SCI, which progressively recover intrinsic, electrophysiological properties to propagate depolarizing signals (D’Amico et al., 2014). Importantly, however, the development of such excitatory feedback loops is also due to a loss of inhibitory inputs.

### Diminished Inhibition

Concurrent with neuroimmune-mediated mechanisms driving enhanced cellular excitability, the neuroimmune system also plays a major role in reducing cellular inhibition following CNS injury. This diminished inhibition, or disinhibition, is largely associated with a loss of inhibitory neurons, reduced concentrations of inhibitory neurotransmitters γ-aminobutyric acid (GABA) and glycine, reduced surface receptor expression, and altered transmembrane ion gradient regulation. The consequence of such compromised GABAergic control results in further enhancement of excitatory activity of circuits throughout the neuraxis. This subsection will, therefore, highlight the crucial neuroimmune factors suggested to drive cellular and synaptic plasticity and subsequent disinhibition.

The initial loss of both glutamatergic and GABAergic neurons following injury is the result of both primary and secondary injury mechanisms, as previously described. Interestingly, various models of central injury utilizing immunohistochemistry have demonstrated increased signaling and connectivity of glutamatergic interneurons over time, whereas the quantity of GABAergic interneurons both proximal and distal to the injury epicenter are persistently decreased for weeks following injury (Meisner et al., 2010; Cantu et al., 2015; Fernández-López et al., 2016; Ueno et al., 2016). Although the initial loss of inhibitory neurons is consequent of microglia/macrophage-mediated apoptosis, this continued, secondary loss of inhibitory neurons is likely due to a loss of inhibitory synaptic tone regulated by neuroimmune factors following injury.

As discussed above, such diminished inhibitory transmission has been attributed to impaired astrocyte-mediated neurotransmitter homeostasis and cytokine-mediated plasticity. Similar to their pivotal role in regulating glutamate homeostasis, astrocytes are crucial for maintaining GABA homeostasis due to their production of the GABA precursor glutamine. Normally, astrocytes convert glutamate and GABA neurotransmitters into glutamine *via* glutamine synthetase, which is then utilized by neurons for further production of either glutamate or GABA (Schousboe et al., 2013). Impaired regulation of this GABA-glutamate-glutamine cycle is observed after CNS injury, as proinflammatory cytokines alter the gene expression of key enzymes involved in this cycle. Specifically, TNFα downregulates glutamate synthetase expression in astrocytes (Zou et al., 2010) and activation of astrocytes and microglia following SCI downregulates the production of the GABA-precursor glutamic acid decarboxylase (GAD_65_; Gwak et al., 2008). Consequently, synthesis and transmission of GABA is thought to be reduced, which is supported by reduced GABA immunoreactivity in astrocytes following SCI (Fernández-López et al., 2014). This may further contribute to an impaired regulation of neural circuits (Um, 2017).

In addition to these biochemical mechanisms of diminished inhibition, neuroimmune mediators are involved in reducing extracellular concentrations of GABA by modulating GABAergic neurotransmission and the expression of vesicular GABA transporters (VGATs). Specifically, neuroimmune mediators prevalent at high concentrations following CNS injury (e.g., TNFα, IL-1β, IL-6, IFNγ) have been shown to reduce the release of GABA and glycine from spinal interneurons and inhibitory descending projections (Vikman et al., 2007; Kawasaki et al., 2008; Grace et al., 2014). Inhibitory neurotransmission is further influenced by IL-10 and microglia, which increase the quantity of VGATs, and IL-1β antagonizes this activity (Lim et al., 2013). Interestingly, increased expression of VGATs following SCI (Gwak and Hulsebosch, 2011; Ko et al., 2018) and stroke (Qian et al., 2018) is suggested to diminish extracellular concentrations of GABA and subsequently reduce inhibitory tone in neural circuits. Based on these findings and the highly sensitive role of astrocytes in mediating neurotransmitter homeostasis, increased astrocytic reuptake of GABA may ultimately increase the synthesis of glutamine and consequently glutamate, thereby further driving neuronal hyperexcitability and circuit plasticity (Mahmoud et al., 2019). However, our understanding of injury-induced VGAT plasticity is far from complete. Seemingly contradictory results suggest VGAT expression is downregulated following CNS injury (Raghavendra Rao et al., 2003; Vemuganti, 2005; Meisner et al., 2010), although these results may be due to an overall decrease in GABAergic signaling and transmission.

In addition to these pre-synaptic mechanisms of plasticity, impaired inhibitory tone following CNS injury is further influenced by neuroimmune factors directly modulating post-synaptic activity. Post-synaptic surface expression of GABA receptors is also altered by cytokine activity, as TNFα causes endocytosis of GABA_A_ receptors (Stellwagen et al., 2005) in *in vitro* assays. However, *in vivo* injury and inflammation models have demonstrated that TNFα and IL-1β increase GABA_A_ receptor trafficking to the cell surface (Serantes et al., 2006; Stück et al., 2012), yet activation of these cytokines has also been shown to suppress post-synaptic GABA receptor activation and decrease inhibitory synaptic strength (Stellwagen et al., 2005; Kawasaki et al., 2008; Pribiag and Stellwagen, 2013; Yan et al., 2015). Therefore, although the quantity of post-synaptic GABA receptors appears to increase, the strength of inhibitory activity is still diminished in a pro-inflammatory environment.

The basis of such dichotomy may be attributed to an overall lack of GABA and glycine production (described previously), but may also be due to the development of a “phenotypic switch” following CNS injury, wherein GABAergic synapses and agonist activation induces an excitatory, depolarizing response rather than hyperpolarizing (Marty and Llano, 2005; Keller et al., 2007; Stück et al., 2012; Huang et al., 2016). In a study by Nabekura et al. (2002), agonist activation of GABA_A_ receptors resulted in elevated intracellular chloride (Cl^−^) concentrations following axotomy and depolarized reversal potential, shifting the balance to favor excitatory post-synaptic potential (EPSP) activation over inhibitory post-synaptic potential (IPSP) activity after injury. Furthermore, the study revealed significantly reduced levels of the neuronal potassium-chloride cotransporter, KCC2, following injury, thereby resulting in dysregulation of the Cl^−^ ion gradient equilibrium and excitatory activity upon agonist binding to GABA receptors (D’Amico et al., 2014; Garraway and Huie, 2016). Since this discovery, models of SCI (Keller et al., 2007; Boulenguez et al., 2010), TBI (Bonislawski et al., 2007; Lizhnyak et al., 2019), and stroke (Jaenisch et al., 2010; Toda et al., 2014) have identified KCC2-mediated switches in GABAergic activity as contributing to impaired inhibitory activity following CNS injury.

Although the exact mechanisms underlying this switch are not yet fully understood, microglia are known to contribute to the downregulation of KCC2. Specifically, models of neuropathic pain after nerve injury have demonstrated that microglia-derived BDNF binds to its neuronal cognate receptor tropomyosin-related kinase B (TrkB), thereby inhibiting *kcc2* mRNA transcription and subsequently decreasing KCC2 membrane insertion following injury (Coull et al., 2005; Ferrini and De Koninck, 2013). Based on this finding, current investigations are beginning to explore the role of neuroimmune mediators in contributing to this GABAergic phenotypic switch. Corradini et al. (2018) found that IL-1β is a modulatory factor involved in reducing KCC2 expression, as the deletion of its cognate receptor IL-1R protected embryos from altered KCC2 and GABA activity. Finally, as injury-induced activation of cytokines and chemokines can engage in a positive pro-inflammatory feedback loop with microglia, factors such as TNFα and IL-1β may be indirectly driving KCC2 downregulation by continuously stimulating the release of microglia-derived BDNF and/or *via* modulating TrkB receptor expression.

In summation, the neuroimmune system is implicated in driving diminished inhibition within neural circuits by reducing the vesicular release of inhibitory neurotransmitters, contributing to weakened synaptic strength, and stimulating a shift in ionic membrane gradients that ultimately impairs sufficient cellular hyperpolarization. Coupled with mechanisms of enhanced excitability (discussed above) and disruptions in neurotransmitter biosynthesis *via* glial GABA-glutamate-glutamine cycle, such presynaptic and postsynaptic plasticity contribute to an imbalanced activation of neural circuits following CNS injury that can have profound physiological implications.

### Synaptic Remodeling

As described in previous sections, injury-induced cell death and axonal degeneration result in a loss of inputs onto spared neurons. Consequently, activated astrocytes and microglia/macrophages not only phagocytose and clear such cellular debris but also contribute to the formation and/or pruning of neuronal synapses and dendritic spines after injury (Blinzinger and Kreutzberg, 1968; Chung et al., 2015; Ziebell et al., 2015). Moreover, hyperactivation of microglia in a chronic inflammatory environment results in excessive pruning, and depletion of these cells has been shown to attenuate spine loss and apoptosis after TBI (Wang et al., 2020). Compared to the primarily phagocytic role of microglia/macrophages, activated astrocytes exhibit a multifunctional role in synaptic remodeling by both promoting the recovery of functional synapses as well as mediating their elimination *via* direct and indirect signaling mechanisms. Although reviewed in more depth previously (Chung et al., 2015; Burda et al., 2016), astrocytes have been shown to directly promote synapse formation *via* expression of the synaptogenic molecules thrombospondin 1 and 2 (TSP-1/2) after injury (Liauw et al., 2008; Tyzack et al., 2014), as well as mediate synapse elimination directly *via* phagocytosis (Chung et al., 2013) and indirectly *via* secretion of transforming growth factor-β (TGF-β), which induces neuronal expression of phagocytic signals recognized by microglia (Stevens et al., 2007).

In addition to these glial-derived neuroimmune mediators, factors such as TNFα and IL-1β have been shown to regulate synaptic remodeling and function. As previously highlighted, these factors are predominantly known for driving cellular plasticity by mediating synaptic receptor expression and synaptic strength; however, growing evidence supports the global role of these cytokines in mediating circuit-wide function. Specifically, glia-derived TNFα is implicated as a key mediator in synaptic scaling, the homeostatic process by which the strength of all synapses on a cell are modulated (Stellwagen and Malenka, 2006; Burda et al., 2016). Moreover, remodeling of cortical dendritic spines following systemic inflammation is suggested to be mediated by TNFα and contributes to deficits in learning and cognitive function (Garre et al., 2017). IL-1β is also among the most notable cytokines contributing to such synaptic plasticity *via* direct and indirect mechanisms of modulating the structure and function of dendritic spines (Li et al., 2003; Gilmore et al., 2004), number of synaptic sites (Mishra et al., 2012), and synapse stabilization (reviewed in more detail in Besedovsky and del Rey, 2011; Pozzi et al., 2018; Rizzo et al., 2018). Furthermore, numerous cytokines are suggested to influence long-term potentiation (LTP) of synaptic transmission between neurons *via* gliogenic paracrine signaling and may, therefore, contribute to the development of pain hypersensitivity and cognitive dysfunction (Besedovsky and del Rey, 2011; Kronschläger et al., 2016). IL-1β, in particular, is suggested to inhibit LTP (Katsuki et al., 1990; Bellinger et al., 1993) and may, therefore, influence cognitive recovery following neurotrauma; however, such findings are controversial due to reported differential effects of endogenous vs. exogenous cytokine activity (Besedovsky and del Rey, 2011). Although the majority of studies examining neuroimmune-mediated synaptic plasticity have focused on the implications in relation to cognitive development, learning, and memory, these findings become increasingly intriguing within the context of CNS injury. As it is well documented that deficits in cognitive and circuit function following TBI (White et al., 2017), stroke (Di Filippo et al., 2008), and SCI (Ferguson et al., 2012) are associated with alterations in LTP, understanding the role of persistent neuroimmune signaling in mediating this activity could elucidate potential targets for modulating synaptic plasticity in affected neural circuits.

Throughout this section, many of the aforementioned neuroimmune factors exhibit multi-functional roles for mediating cellular and synaptic plasticity that ultimately produce overlapping implications. For example, TNFα activity is repeatedly identified as a crucial factor that modulates glutamatergic and GABAergic receptor expression to enhance excitation and diminish inhibition but also modulates synaptic scaling and circuit-wide synapse activity. Although it could be suggested that such synaptic plasticity will further enhance the excitability of neural circuits, further research is necessary to draw this conclusion.

## Neuroimmune-Mediated Axonal and Anatomical Plasticity

Following an injury to the CNS, anatomical plasticity is observed throughout the neuraxis including cortical, subcortical, and intraspinal levels. Specifically, such plasticity includes afferent fiber sprouting and reorganization of cortical and subcortical structures following CNS injury. The basis for this anatomical plasticity is at least partially attributed to plasticity in cellular and synaptic activity described previously. As enhanced cellular excitability alters cell-to-cell signaling activity after an injury, structural processes undergo diverse mechanisms of plasticity that ultimately remodel entire neural circuits throughout the neuraxis. Moreover, these structural changes further influence cellular activity and synaptic plasticity, establishing a feedforward loop of plasticity that ultimately influences functional outcomes following CNS injury. This section will, therefore, highlight various structural changes observed after injury and the mechanisms by which the neuroimmune system drives such plasticity.

### Axonal Sprouting and Remodeling

Changes in cellular and synaptic activity are not the only forms of plasticity observed after injury. Growth of spared injured and un-injured axons following CNS injury is a well-documented phenomenon that is suggested to aid in the formation of new connections and recovery of circuit activity after injury. Although various forms of growth are proposed to exist depending on origin (e.g., collateral sprouting vs. frank regeneration), it is quite evident that such structural and anatomical plasticity occurs throughout the neuraxis. Sprouting of ascending and descending spinal pathways, propriospinal neurons, and subcortical pathways have been demonstrated in various models of CNS injury and are thought to contribute to spontaneous functional recovery or maladaptive dysfunction after injury, depending upon the circuit being assessed (Raineteau and Schwab, 2001; Wieloch and Nikolich, 2006; Tuszynski and Steward, 2012; Carmichael et al., 2017; O’Shea et al., 2017; Farrell et al., 2019; Michael et al., 2019).

Reactive astrocytes are implicated as crucial neuroimmune cells involved in axonal sprouting and reorganization of cortical, subcortical, and spinal networks following injury. As previously described, reactive astrocytes have been historically regarded as an inhibitory obstacle that impedes axonal sprouting and growth after injury. This is exemplified by findings that reduced astrocytic reactivity is correlated with improved serotonergic fiber growth into the contralateral, denervated spinal cord following a hemisection SCI (Giménez Y Ribotta et al., 1995). Furthermore, this growth-inhibitory activity of reactive astrocytes is supported by evidence that these cells express a myriad of inhibitory molecules such as CSPGs (e.g., brevican, neurocan, versican, phosphacan) which inhibit axonal sprouting and consequently influence tissue remodeling and reorganization following injury (McKeon et al., 1991; Anderson et al., 2016). Numerous studies utilizing transgenic approaches to attenuate or ablate astrocyte activity have also demonstrated increased fiber growth and axonal plasticity following CNS injury (Bush et al., 1999; Menet et al., 2000, 2003; Sofroniew, 2005). However, contrary to the premise that astrocytes exert an inhibitory function in mediating axonal plasticity, recent evidence demonstrates that attenuation or ablation of reactive astrocytes following CNS injury does *not* promote axonal growth (Anderson et al., 2016), suggesting a growth-supportive role of these glial cells post-injury. Indeed, numerous studies have highlighted reactive astrocytes as supportive cells for axon growth. Specifically, studies have suggested that immunoreactive astrocytes are crucial for promoting fiber sprouting and growth in limbic and striatal cortical circuits following CNS injury (Gage et al., 1988; Kawaja and Gage, 1991). Consistent with this evidence, astrocyte-associated fibronectin is implicated as an important substrate for promoting CNS axon growth (Tom et al., 2004). Moreover, astrocytic TSP-1/2 is implicated in contributing to compensatory axonal sprouting in models of stroke injury, as TSP-1/2 knockout mice exhibit significantly less cortical and striatal sprouting as well as impaired behavioral recovery (Liauw et al., 2008). Taken together, these contrasting studies not only highlight the complex functions of reactive astrocytes but also exemplify the importance of further research exploring this topic. Furthermore, although this seemingly contradictory evidence is currently a matter of great debate and intrigue, the differential functional role of reactive astrocytes may be attributed to variances in injury/animal models, experimental time points, the multitude of factors expressed by reactive astrocytes, and heterogeneous forms of activated astrocytes following CNS injury.

In addition to the production of growth-supportive and -inhibitory factors to influence axonal plasticity post-injury, neuroimmune cells also produce neurotrophic factors that can similarly mediate axonal sprouting and regrowth. Moreover, neuroimmune-derived neurotrophic factors interact with immune mediators such as cytokines and chemokines to influence immune cell activation and establish an interconnected system for driving axonal plasticity (reviewed in more detail in Peruzzotti-Jametti et al., 2014; Garraway and Huie, 2016; Liberman et al., 2018). This interconnected regulatory system is exemplified by findings that TNFα specifically increases BDNF production in microglia/macrophages (Schulte-Herbrüggen et al., 2005) and increases astrocyte production of NGF and GDNF (Kuno et al., 2006). Importantly, each of these neurotrophic factors is implicated as key factors involved in promoting axonal growth post-injury. Based on this, persistently elevated neuroimmune cell and proinflammatory cytokine activity is suggested to drive neurotrophic factor expression and consequently axonal plasticity.

The physiological implications of this network are exemplified by NGF/TrkA signaling-induced sprouting. Injury-induced up-regulation of NGF expression results in enhanced binding to TrkA receptors on primary nociceptive calcitonin gene-related peptide (CGRP)-expressing afferent fibers. Importantly, NGF administration *in vivo* has been shown to increase the sprouting of TrkA^+^ fibers (Mearow, 1998; Mantyh et al., 2011). As nearly all TrkA^+^ neurons express CGRP (Averill et al., 1995), this increased sprouting and enhanced synthesis of CGRP and nociceptive ion channel expression (e.g., TRPV1) ultimately enhances nociceptive signaling and sensitivity (Mantyh et al., 2011). Moreover, sprouting and arborization of TrkA^+^ CGRP^+^ fibers are strongly enhanced following CNS injury and plays a role in the remodeling of sensory and sympathetic fibers implicated in the development of neuropathic pain and autonomic dysreflexia (Krenz et al., 1999; Cameron et al., 2006; Mantyh et al., 2011; Nitzan-Luques et al., 2013; Mironets et al., 2018, 2020). A key role of this signaling in such plasticity was corroborated by studies demonstrating that blocking NGF/TrkA signaling attenuates CGRP^+^ afferent sprouting after injury (Christensen and Hulsebosch, 1997; Krenz et al., 1999; Mantyh et al., 2011).

Interestingly, TNFα/TNFR1 signaling is implicated as a critical neuroimmune mediator of afferent sprouting by modulating the sensitivity of TrkA signaling to NGF (Wheeler et al., 2014; Mironets et al., 2018). Moreover, central inhibition of soluble-TNFα *via* continuous intrathecal delivery of the biologic XPro1595 following SCI has been shown to significantly decrease TrkA expression below-injury and attenuate CGRP^+^ fiber sprouting (Mironets et al., 2018, 2020). Coupled with findings that inhibition of TNFα increased lumbar NGF levels (Mironets et al., 2018) and that NGF is primarily expressed by non-neuronal cells post-injury (Krenz and Weaver, 2000), these results suggest that TNFα/TNFR1 signaling mediates neuronal TrkA receptor sensitivity to NGF and drives subsequent afferent sprouting and plasticity. In addition to mediating sensory afferent sprouting, TNFα signaling also contributes to collateral sprouting of corticospinal tract (CST) fibers and influences locomotor recovery after TBI (Oshima et al., 2009).

Numerous studies have demonstrated a crucial role for immune-mediated activation of other neurotrophic factors and modulatory cytokines to promote axonal plasticity after CNS injury. In addition to TNFα, cytokines such as IL-6 and IL-1β contribute to axonal plasticity and mediate growth factor release post-injury. Specifically, IL-6 is proposed to support axonal growth and plasticity post-injury, as genetic depletion of this cytokine reduces serotonergic fiber growth post-injury (Ramer et al., 1998; Cafferty et al., 2004). In contrast, IL-1β is proposed to suppress axonal plasticity as recombinant IL-1β administered to transgenic IL-1β knockout mice reduced the quantity of CST fibers and worsened locomotor functional outcomes (Boato et al., 2013). Moreover, the absence of IL-1β resulted in increased CST sprouting and improved functional outcomes. Interestingly, however, the role of IL-1β in axonal plasticity may not be solely inhibitory, as studies suggest that IL-1β promotes the release of growth-supportive factors such as IL-6 and NGF by stimulating reactive astrocytes (Lindholm et al., 1987; Cotman, 1999). IL-1β signaling is also suggested to interfere with BDNF/TrkB signaling (Tong et al., 2008, 2012) and NT-3 signal transduction (Soiampornkul et al., 2008). As BDNF and NT-3 are both critically involved in axonal growth, sprouting, and plasticity (Chen et al., 2008; Garraway and Huie, 2016), IL-1β may be a crucial mediator of growth-supportive and -inhibitory axon plasticity following CNS injury.

### Phenotypic Plasticity

As synapses and axons undergo strengthening, pruning, and sprouting, this plasticity may contribute to the additional expansion of axonal arbors and the formation of new connections that influence circuit signaling in a newfound capacity. Within the context of SCI, this is commonly exemplified by extensive arborization of sensory afferents in the deep dorsal horn (laminae III–V) proximal and distal to the injury epicenter. Normally, touch-sensitive Aβ-fibers within the DRG transmit innocuous, non-noxious cutaneous information and synapses in laminae III–V of the spinal dorsal horn (Todd, 2010). After an injury, Aβ-fibers sprout dorsally into superficial laminae I–II, wherein mechanonociceptive C-fibers and mechanothermal Aδ afferents normally terminate and transmit noxious, nociceptive sensory signals (Woolf et al., 1992; Lekan et al., 1996; Todd, 2010). Nociceptive Aδ- and C-fibers are also suggested to exhibit sprouting into deeper laminae (III-V) following SCI (Krenz and Weaver, 1998b; Ondarza et al., 2003), thereby further expanding primary nociceptive terminal fields into regions typically associated with non-painful touch stimuli. Although these findings remain somewhat controversial due to lack of tracer specificity (Bao et al., 2002; Shehab et al., 2003), there is evidence to suggest that Aβ-fibers drive activation of lamina II nociceptive neurons (Kohno et al., 2003; Woodbury et al., 2008). Consequent of such Aβ-, Aδ-, and C-fiber sprouting, spinal circuits undergo enormous remodeling that contributes to a loss of segregation between touch-specific lamina (i.e., deeper laminae) and pain-specific lamina (i.e., superficial laminae) within the spinal cord (described in more detail in Kuner and Flor, 2016).

In addition to this structural remodeling of spinal circuits, neurons undergo genomic changes that likely contribute to not only synaptic and axonal plasticity but also “phenotypic shifts.” Normally, cells are identified *via* known neuropeptide markers, morphology, and/or electrophysiological characteristics. After an injury, however, many of these defining characteristics are altered. Within spinal sensory circuits, for example, nociceptive Aδ- and C-fiber neurons are commonly identified by their molecular expression of CGRP, substance P (SP), and/or TrkA, whereas non-nociceptive Aβ neurons typically do not express these markers (Navarro et al., 2007; Castaneda-Corral et al., 2011). After an injury, Aβ-fiber cells exhibit *de novo* CGRP and SP expression, as well as a shift in electrical and neurochemical activity (e.g., spontaneous ectopic discharge, enhanced excitability; reviewed in more detail in Molander et al., 1994; Bester et al., 2000; Navarro et al., 2007; Devor, 2009; Hou et al., 2009; Latremoliere and Woolf, 2009; Nitzan-Luques et al., 2013). Consequently, activation of these normally innocuous stimuli-encoding afferents by low-intensity stimuli (e.g., light-touch) may result in the transmission of nociceptive signals. Moreover, this shift in the phenotypic expression of nociceptive neuromodulators may contribute to further expansion of nociceptive terminal fields, even in the absence of afferent sprouting, and further drive central pain circuit activity. Together, this plasticity is thought to be a mechanism underlying the development of neuropathic pain after injury (Devor, 2009; Kuner and Flor, 2016).

Phenotypic switches are also suggested to affect Aδ- and C-fiber afferents innervating the lower urinary tract (LUT) following SCI. Within the intact micturition reflex pathway, Aδ-fibers are considered to be essential for generating storage and voiding reflexes and C-fibers primarily respond to noxious stimuli (e.g., bladder inflammation; Häbler et al., 1990; Fowler et al., 2008). After SCI, C-fibers are suggested to adopt a mechanosensitive phenotype as these fibers predominantly initiate voiding rather than Aδ afferents (De Groat and Yoshimura, 2010). This finding is based on data demonstrating that the C-fiber neurotoxin capsaicin blocks voiding after SCI, but not in intact spinal cord models (De Groat et al., 1990; Cheng et al., 1999; De Groat and Yoshimura, 2012). These findings also correlate with clinical data demonstrating increased expression of the capsaicin-sensitive receptor TRPV1 in suburothelial nerves of people with bladder overactivity (i.e., neurogenic detrusor overactivity) following various lesions and injuries to the spinal cord (Brady et al., 2004; Apostolidis et al., 2005; De Groat and Yoshimura, 2010). As TRPV1 is considered to be predominantly expressed by C-fibers, these findings suggest that heightened C-fiber activity contributes to abnormal bladder activity post-injury. Moreover, increased expression and expansion of C-fiber-associated neuropeptides, such as VIP, IB4, and PACAP, following SCI indicates sprouting of spinal C-fibers and is correlated with recovery of micturition reflexes (Morgan et al., 1999; Vizzard, 2006; Zinck and Downie, 2008; De Groat and Yoshimura, 2010). Although each of these studies predominantly highlight phenotypic switches associated with C-fibers, evidence from peripheral nerve injury models suggest that spinal Aδ-fiber afferents undergo molecular changes post-injury (Ji et al., 2007; Navarro et al., 2007; Ruscheweyh et al., 2007) and may, therefore, contribute to micturition circuit remodeling. As some Aδ-fibers normally express TRPV1 (Mitchell et al., 2010) and peripheral injury results in upregulation of sensory neuron TRPV1 expression (Ramer et al., 2012) as well as mechanothermal plasticity of Aδ-fibers (Ji et al., 2007), phenotypic switches within this afferent group may obfuscate the functional role of Aδ-fibers post-injury.

Although the mechanisms underlying these phenotypic and genomic changes are not yet fully understood, inflammation is known to modulate receptors and ion channels in peripheral sensory terminals, which can result in cellular plasticity (i.e., enhanced excitation, diminished inhibition) and structural plasticity (i.e., afferent sprouting and reorganization) in the spinal cord (Grace et al., 2014; Kuner and Flor, 2016). Furthermore, neuroimmune-derived neurotrophins and proinflammatory cytokines are implicated as contributing to sensory afferent phenotypic switches. Notably, nerve injury and inflammatory pain models demonstrate that large-diameter sensory neurons (i.e., Aβ afferents) begin expressing BDNF (Zhou et al., 1999; Ohtori et al., 2002), a pronociceptive factor normally released by C-fibers (Garraway and Huie, 2016). Additionally, as NGF and TNFα signaling are known to increase CGRP expression (Lindsay and Harmar, 1989; Krenz and Weaver, 1998b; Bowen et al., 2006) and are persistently heightened post-injury (Mironets et al., 2018), these neuroimmune factors may also contribute to driving *de novo* CGRP expression in Aβ-fibers.

### Cortical, Subcortical, and Spinal Reorganization

Structural and functional changes throughout the brain, brainstem, and spinal cord following injury also impact motor movements and sensory perceptions (Raineteau and Schwab, 2001; Kuner and Flor, 2016). Various models of CNS injury have demonstrated extensive cortical rewiring and alterations in motor and sensory cortical territories, as regions representative for intact targets progressively expand into deafferented regions (Nudo and Milliken, 1996; Mohammed and Hollis, 2018). Coupled with the growth of new horizontal axons and arbor expansion, as well as overall plasticity in cellular physiology and activity, this cortical plasticity ultimately reshapes entire neural circuits and contributes to both adaptive and maladaptive functional outcomes. For instance, central and peripheral sensitization and increased spontaneous nociceptive activity after injury may contribute to the reorganization of cortical and subcortical regions associated with processing the sensory, emotional, and cognitive components of pain. Specifically, SCI is associated with reduced gray matter in the anterior cingulate cortex (ACC), insula, somatosensory cortex, and regions of the brainstem compared to non-SCI individuals (Jutzeler et al., 2016). Moreover, the development of neuropathic pain in SCI individuals was associated with the magnitude of cord atrophy. Injury-induced neuropathic pain is also associated with structural and functional plasticity within the amygdala, ACC, hippocampus, prefrontal cortex, and primary somatosensory cortex (S1; Endo et al., 2007; Wrigley et al., 2009; Mole et al., 2014; Jutzeler et al., 2016; reviewed in more detail in Kuner and Flor, 2016; Yang and Chang, 2019). In addition to these findings, chronic neuropathic pain is suggested to shift cortical pain circuit activity from nociceptive circuit activation to emotional circuit activation over time, as individuals with chronic back pain engage emotional circuits (i.e., amygdala, hippocampus, orbitofrontal cortices, PFC) in response to pain stimuli, whereas individuals with subacute back pain engage regions involved in nociceptive sensory processing (i.e., insula, thalamus, midbrain, ACC, S1; Hashmi et al., 2013). Interestingly, reorganization of the sensory cortical map is associated with upregulation of BDNF as early as 1-day post-SCI (Endo et al., 2007), and is implicated as a critical mediator involved in processing emotional aspects of neuropathic pain within the cortex (Thibault et al., 2014).

In contrast to this maladaptive consequence of reorganization, cortical reorganization and anatomical plasticity throughout the neuraxis are critically involved in the functional recovery of learned motor movements and fine-tuning of dexterous skills (Lynskey et al., 2008; Mohammed and Hollis, 2018). For instance, functional improvements in weight support and locomotion following SCI and combined pharmacological and rehabilitative strategies correlated with expansion of the trunk motor cortex and increased sprouting of corticospinal axons rostral to the lesion site (Manohar et al., 2017). Furthermore, functional reorganization of the somatosensory cortex was also observed, suggesting an integrative and synergistic role of these cortical regions in mediating sensorimotor circuit recovery after injury (Farrell et al., 2019). Functional motor plasticity is also attributed to the reorganization of brainstem centers and spinal segment levels following injury-induced disruption of descending supraspinal tracts such as the rubro-, vestibulo-, reticulo-, and tectospinal tracts (Li, 2017).

Interestingly, heightened spinal reorganization temporally correlates with spinal expression of TNFα, IL-6, and NT-3 in a model of stroke injury (Sist et al., 2014). This study further suggested that motor-related plasticity occurs in a finite temporal window and that this window of spontaneous recovery is related to the expression of inflammatory cytokines and neurotrophic factors. Coupled with previously described findings regarding the role of TNFα signaling in CST regeneration and how TNFα and IL-6 contribute to cellular and synaptic plasticity, it is evident that these cytokines contribute to driving reorganization of motor circuits post-injury. In addition to these factors, BDNF/TrkB signaling contributes to motor cortex plasticity following TBI (Ueno et al., 2012), promotes the regeneration of supraspinal motor tracts (Jin et al., 2002), and is implicated as a crucial signal for initiating structural plasticity and anatomical reorganization (Sist et al., 2014). As each of these factors are intimately involved in a feedforward autocrine cycle with the neuroimmune system, developing research into the role of immune cells in anatomical reorganization will provide insight for manipulating this plasticity.

Injury-induced reorganization also affects subcortical and spinal autonomic circuits, eliciting adaptive and/or deleterious outcomes. Rewiring of medullary respiratory networks, sprouting of serotonergic fibers and spinal interneurons, and formation of new neural connections are key forms of anatomical plasticity that positively influence respiratory recovery after CNS injury (Bezdudnaya et al., 2017; Warren et al., 2018; Zholudeva et al., 2018). Additionally, rewiring local intraspinal networks can contribute to the recovery of micturition reflexes in rodent models of SCI (De Groat and Yoshimura, 2012; Hou and Rabchevsky, 2014). However, over time, such intraspinal anatomical plasticity can be detrimental, as seen with the development of neuropathic pain (described above), autonomic dysreflexia, and detrusor-sphincter dyssynergia. For instance, within the intact nervous system, descending modulatory inputs from supraspinal vasomotor neurons within the brainstem synapse onto sympathetic preganglionic neurons (SPNs) throughout the thoracolumbar cord (~T1-L2) and/or parasympathetic preganglionic neurons (PPNs) in the sacral cord (~S2-S4) to ultimately influence functional activity in the periphery. The activity of SPNs and PPNs is therefore primarily mediated by supraspinal control centers and propriospinal interneurons relaying primary afferent activity from the periphery (reviewed in more detail in Fowler et al., 2008; Eldahan and Rabchevsky, 2018). Following SCI, supraspinal projections are often lost, resulting in these autonomic circuits to be solely regulated by spinal interneurons. Thus, in addition to mechanisms of cellular and axonal plasticity contributing to increased activation of spinal neurons, anatomical reorganization and plasticity of propriospinal fibers is observed within the dorsal gray commissure and dorsal horn and is attributed to the expansion of spinal segment innervation post-injury (De Groat et al., 1981; Krassioukov et al., 2002; Hou et al., 2008; Ueno et al., 2016; Michael et al., 2019). The occurrence of this plasticity is at least partially attributed to increased BDNF and NGF in the CNS, as well as upregulated expression of neurotrophic and inflammatory factors by peripheral organs such as the bladder (De Groat and Yoshimura, 2012; Gonzalez et al., 2014). Expression of BDNF and NGF by sympathetic and parasympathetic afferents also contributes to regulating autonomic output to peripheral organs, and may therefore further modulate neuroimmune activity and associated plasticity (Zaidi et al., 2005; Mattson and Wan, 2008).

Large-scale reorganization of anatomical regions and circuits is the combined outcome of plasticity occurring at cellular, synaptic, and axonal levels which cumulatively influence functional outcomes. Current research efforts have sought to therapeutically target cortical, subcortical, and intraspinal plasticity to modulate the development and/or severity of these physiological consequences. This includes rehabilitative training and electrical stimulation to promote strength and motor recovery (Li, 2017); pharmacotherapies for modulating sensitization and pain signaling (Colloca et al., 2017); and primarily palliative measures for the management of respiratory and autonomic conditions following CNS injury (Galeiras Vázquez et al., 2013; Hou and Rabchevsky, 2014). However, as the neuroimmune system has steadily emerged as a crucial mediator involved in driving plasticity at each of these levels, current and future therapeutic strategies have focused on targeting this system.

## Functional and Therapeutic Implications

As discussed throughout this review, neuroimmune-mediated plasticity greatly contributes to alterations in neural circuit activity following neurotrauma. As researchers continue to investigate the functional implications of this plasticity, emerging evidence further supports the crucial role of the neuroimmune system in driving plasticity and physiological outcomes after CNS injury. Specifically, neuroimmune factors mediate cellular, structural, and anatomical plasticity that contribute to functional changes post-injury. Moreover, such studies have highlighted the importance of investigating the anatomical level of the CNS at which plasticity occurs, as this greatly influences the functional outcomes. For example, it is suggested that post-stroke motor recovery and spasticity are associated with different anatomical regions of plasticity, as efforts focus on targeting cortical plasticity to promote motor recovery and modulating reticulospinal tract hyperexcitability to manage spasticity and abnormal motor synergy (Li, 2017; Li et al., 2019). Therefore, although this section will broadly highlight the functional roles and therapeutic implications of key neuroimmune mediators involved in injury-induced plasticity, it is important to explore the injury level, severity, and region of interests in relation to specific functional and therapeutic outcomes.

### Functional Implications of Neuroimmune-Mediated Plasticity

Within sensory circuits, the impact of neuroimmune-mediated cellular plasticity is observed as an enhanced transmission of pain and sensory signals. Physiologically, such plasticity is necessary and adaptive for inducing reflexive responses to stimuli independent of cortical control; however, over time, this enhanced sensory transmission can lead to the development of neuropathic pain, a neuroimmune disorder characterized by heightened sensitivity to noxious and innocuous stimuli. As previously outlined by Watkins and colleagues (Grace et al., 2014), central and peripheral immune signaling is integral to normal pain processing and such neuroimmune and inflammatory mediators directly mediate nociceptive pain neuron activity (Sommer and Kress, 2004; Binshtok et al., 2008; Costigan et al., 2009). Following injury, heightened levels of immune and inflammatory mediators persistently activate peripheral terminals and central nociceptive neurons, resulting in increased sensory and pain transmission. Coupled with mechanisms for enhancing excitation and diminishing inhibition, cytokines such as TNFα (Leung and Cahill, 2010; Zhang et al., 2011), IL-1β (Sweitzer et al., 1999; Zelenka et al., 2005), and IL-6 (DeLeo et al., 1996; Arruda et al., 2000) contribute to pain pathogenesis *via* direct modulation of nociceptor activity (for detailed reviews highlighting the role of cytokines in neuropathic pain development, see Zhang and An, 2007; Leung and Cahill, 2010; Ellis and Bennett, 2013). Specifically, patch-clamp electrophysiology from lamina II neurons of the spinal dorsal horn demonstrate that TNFα enhances cellular activation by increasing the frequency of spontaneous EPSCs and enhancing AMPA- and NMDA-induced currents (Kawasaki et al., 2008; Zhang et al., 2011); IL-1β increases the frequency and amplitude of spontaneous EPSCs and enhances NMDA-induced currents; and both IL-1β and IL-6 suppress GABAergic and glycinergic currents and reduce the frequency of spontaneous IPSCs (Kawasaki et al., 2008). Together, such enhanced excitation is proposed to contribute to driving afferent sprouting and anatomical reorganization (e.g., phenotypic switch of Aβ-fibers; Aδ and CGRP sprouting; reorganization of sensorimotor cortices) which ultimately drives sensory circuits that reinforce neuropathic pain signaling. Furthermore, such cytokine-mediated plasticity is proposed to engage in a feed-forward proinflammatory cycle of plasticity by further activating cortical and spinal microglia/macrophages, which are implicated as key cells involved in afferent sprouting, synaptic plasticity, and reorganization of cortical sensory circuits (Inoue and Tsuda, 2018; Zhou et al., 2019) as well as crucial for regulating higher-order processing of nociceptive signals (Zhao et al., 2007; Beggs and Salter, 2010). As a consequence of persistent microglia/macrophage activation, proinflammatory cytokines and chemokines continue to be produced and thereby establish a chronic, inflammatory loop which ultimately exacerbates and solidifies plasticity in the sensory circuit.

The imbalance in excitatory to inhibitory transmission observed within sensory neural circuits following injury further interweaves motor and autonomic circuits, as sensory afferent neurons projecting from the periphery form monosynaptic and polysynaptic connections with motor (i.e., somatic and autonomic) efferent neurons in the spinal cord and modulate output to skeletal muscles and visceral organs, thereby mediating locomotive and autonomic output. Therefore, enhanced activation of sensory neurons following stimulation, coupled with diminished inhibitory activity by GABAergic interneurons, results in hyperactivation of motoneurons. Indeed, motoneurons within the spinal ventral horn exhibit reduced IPSPs and increased, prolonged EPSPs in response to a brief sensory stimulus following SCI (Baker and Chandler, 1987; D’Amico et al., 2014). Moreover, such motoneurons exhibit intrinsic properties of hyperexcitability, such as plateau potentials caused by persistent Na^+^ and Ca^2+^ currents, indicative of enhanced excitatory activity (Hounsgaard and Kiehn, 1985; D’Amico et al., 2014).

Functionally, this enhanced excitability of motoneurons is a powerfully adaptive feat as synaptic plasticity throughout the neuraxis can promote recovery of locomotion independent from supraspinal regulation. Additionally, enhanced cellular excitability following injury contributes to the recovery of spontaneous micturition and autonomic reflexes (De Groat and Yoshimura, 2012). However, such plasticity within these sensorimotor circuits may also result in maladaptive functional outcomes, such as spasticity, hyperreflexia, and autonomic dysreflexia (Baker and Chandler, 1987; Calancie et al., 1996, 2002; Krenz and Weaver, 1998a; Krassioukov et al., 2002; D’Amico et al., 2014; Walters, 2018).

The neuroimmune system has been implicated in driving other examples of cellular plasticity within the motor and autonomic circuits (Llewellyn-Smith et al., 1997; D’Amico et al., 2014; Hou and Rabchevsky, 2014). For instance, following SCI, there is increased recruitment of sympathetically-associated glutamatergic propriospinal interneurons into the spinal sympathetic reflex (SSR) circuit and increased glutamatergic synapses onto sympathetic preganglionic neurons (Llewellyn-Smith et al., 1997; Ueno et al., 2016). These changes are proposed to drive hyperactivation of this system (i.e., sympathetic hyperreflexia) and contribute to the development of autonomic dysreflexia and dysimmunity (Michael et al., 2019; Mironets et al., 2020). Infiltration of monocyte-derived macrophages *via* MMP-9 within the lumbar spinal cord also increase GABAergic activity within locomotor networks and impairs recovery of motor function after SCI (Hansen et al., 2013, 2016). This is similarly supported by studies by Kaspar and colleaguess (Frakes et al., 2014) demonstrating that microglia can induce motoneuron death *via* NF-κB signaling, and subsequently alter circuit activation properties within chronic inflammatory environments such as CNS injury. These findings further demonstrate that the neuroimmune system is a crucial factor involved in driving cellular plasticity throughout the neural axis and an important area of research for elucidating plasticity within a variety of circuits.

### Therapeutic Targeting of the Neuroimmune System

Advancements in understanding the functional implications of certain neuroimmune factors have greatly contributed to the development of immunomodulatory therapies used to promote beneficial outcomes and suppress maladaptive outcomes. This is exemplified by studies targeting the neuroimmune system to modulate neuropathic pain development. Microglia/macrophages, in particular, are highlighted as “indispensable” neuroimmune cells involved in modulating the developmental onset and severity of neuropathic pain after injury (Detloff et al., 2008; Chhaya et al., 2019). Based on this, numerous studies have explored targeting microglia/macrophages, as ablation or conditional deletion of spinal microglia has been shown to attenuate CGRP^+^ afferent sprouting and LTP-induced mechanical allodynia (Zhou et al., 2019). Thus, therapeutic targeting of microglia/macrophages and related neuroimmune factors to modulate injury-induced plasticity and mitigate neuropathic pain development is an intriguing area of research currently being explored. Indeed, immunomodulatory-based pharmacotherapies are among the predominant strategies explored for the treatment of injury-induced neuropathic pain (Grace et al., 2014; Cavalli et al., 2019), and many of these therapies are known to target microglia (for a review of these treatments, see Grace et al., 2014).

Among such intriguing therapies is the tetracycline antibiotic minocycline that is already in clinical use. In addition to its antibacterial properties, minocycline has been shown to inhibit microglial activation and suppress the upregulation and release of IL-1β and TNFα, as well as provide anatomical neuroprotection following CNS injury and attenuates neuropathic pain (Garrido-Mesa et al., 2013; Ma et al., 2015; Wang et al., 2017; Afshari et al., 2018). Moreover, results from a phase II clinical trial of minocycline treatment after acute SCI demonstrate significant functional improvements in motor function (Casha et al., 2012). Coupled with recent findings that minocycline treatment preserves sympathoexcitatory axons and attenuates the severity of autonomic dysreflexia following SCI (Squair et al., 2018), the use of this immunomodulatory pharmacotherapy to modulate injury-induced plasticity exemplifies the tremendous potential of therapeutically targeting the neuroimmune system. However, further research is necessary to explore these implications on plasticity throughout the neural axis. Understanding the effects of minocycline on LTP or afferent sprouting, for example, could elucidate the potential therapeutic actions of this drug for multiple systems.

Other immunomodulatory therapies such as IL-10 administration (Knoblach and Faden, 1998; Bethea et al., 1999; Hellenbrand et al., 2019), IL-1 receptor antagonists (Akuzawa et al., 2008; McCann et al., 2016), and IL-6/IL-6R signaling inhibition (Mukaino et al., 2010; Yang et al., 2013) have been shown to improve locomotor function after injury. Numerous studies utilizing antibodies selective against immune cell activation and infiltration have also demonstrated improved anatomical plasticity and functional recovery in multiple systems (reviewed in more detail in Trivedi et al., 2006). Notably, inhibition of neutrophil and macrophage infiltration by blocking the CD11d/CD18 integrin has been shown to not only reduce chronic pain but also improve motor function and reduce the severity of autonomic dysreflexia events (Gris et al., 2004). These functional outcomes were further correlated with improved morphological, cellular, and anatomical outcomes, thereby supporting the influential role of neuroimmune-mediated tissue recovery and functional outcome. However, replication of this study by Dietrich and colleaguess (Hurtado et al., 2012) did not support these findings, as anti-CD11d monoclonal antibody treatment resulted in non-significant improvements in motor activity and tissue sparing, but continued mechanical allodynia. In a follow-up commentary, Weaver et al. (2012) commented that the variability in these outcomes may be attributed to differences in methodologies, control baselines, and subjective behavioral measures. Nevertheless, these studies highlight the importance of assessing such immunomodulatory therapies in different models of CNS injury.

The widespread and interconnected implications of neuroimmune-mediated plasticity become further apparent when assessing injury-induced plasticity within autonomic circuits. As previously described, sprouting of sensory afferents within the spinal cord are thought to increase the activation of intraspinal interneurons and further drive intraspinal and/or cortical circuit reorganization. For instance, within the SSR circuit, intraspinal CGRP^+^ sprouting is proposed to increase the activation of propriospinal interneurons and drive the development and intensification of sympathetic hyperreflexia over time (Eldahan and Rabchevsky, 2018). TNFα has been implicated as a key factor involved in driving CGRP^+^ sprouting by mediating NGF/TrkA signaling and influences activity and plasticity throughout the neuraxis, including the autonomic nervous system (Hermann and Rogers, 2008; Kisiswa et al., 2013) and SSR circuit. In a series of studies conducted by Mironets et al. (2018, 2020), pharmacological inhibition of soluble TNFα *via* central administration of the biologic mimetic XPro1595 attenuated this injury-induced sprouting and reduced the recruitment of interneurons within the SSR circuit. This effect on anatomical plasticity was further correlated with attenuated sympathetic hyperreflexia, as indicated by the mitigation of autonomic dysreflexia and ensuing peripheral immune dysfunction.

Interestingly, central inhibition of soluble-TNFα *via* XPro1595 also improves motor function and reduces lesion size after SCI (Novrup et al., 2014). This improvement in locomotor function is supported by findings that XPro1595 significantly promotes axonal remyelination and particularly large motor fibers (Brambilla et al., 2011). Similar functional and physiological improvements were observed following XPro1595 treatment in a stroke injury model (Clausen et al., 2014), and was shown to modulate synaptic strength and plasticity in the hippocampus (Sama et al., 2012). Based on this body of literature, it is abundantly clear that injury-induced activation of the neuroimmune system and the resulting increase in cytokines, such as TNFα, contribute to widespread plasticity that influences the functional activity of numerous neural circuits. Furthermore, the use of immunomodulatory therapies to improve functional recovery may have synergistic effects in other circuits throughout the neural axis, resulting in an array of outcomes that may be beneficial and/or deleterious depending on the context. For instance, systemic administration of XPro1595 is suggested to exacerbate depressive phenotypes following SCI (Farrell and Houle, 2019), supporting previous findings that central but not systemic XPro1595 administration is therapeutically beneficial (Novrup et al., 2014). Although intracerebroventricular XPro1595 administration was also ineffective in treating SCI-associated depression, it did increase the behavioral expression of anhedonia (Farrell and Houle, 2019), further demonstrating the complexity of neuroimmune signaling post-injury.

Rehabilitation is an intriguing means to alter neuroimmune activity non-pharmacologically and promote functional recovery. Rehabilitative strategies are among the principal therapies initiated after neurotrauma and focus on influencing plasticity to promote recovery of lost or impaired functions. Commonly used strategies include passive and/or active exercise, electrical stimulation, and retraining of skills such as locomotion, speech, and cognitive function (Lynskey et al., 2008; Turolla et al., 2018). Numerous studies have demonstrated the cellular, molecular, and anatomical plasticity effects of these rehabilitative strategies, but emerging evidence has supported a role for the neuroimmune system in mediating this plasticity. Specifically, initiation of daily aerobic exercise beginning 5 days after SCI in a rodent model reduces nociceptive afferent sprouting in the dorsal horn and attenuates the infiltration and activation of macrophages and microglia in the spinal cord (Detloff et al., 2014; Chhaya et al., 2019). Models of TBI and stroke have also demonstrated that exercise attenuates microglial activation and shifts microglial polarization from proinflammatory M1 toward anti-inflammatory M2 (Piao et al., 2013; Jiang et al., 2017). These findings correlate with increased expression of IL-10 and neurotrophic factors BDNF and IGF-1, as well as improved cognitive function. Importantly, neurotrophic factors, including BDNF, form an autocrine signaling feedback loop with the neuroimmune system by interacting with pro-inflammatory cytokines, such as TNFα and IL-1β, and stimulate microglial activation (Tong et al., 2008; Zhang et al., 2014; Xie et al., 2017). Moreover, increased expression of BDNF following CNS injury is suggested to be primarily derived from glial cells associated with the neuroimmune system—particularly microglia and astrocytes (Pöyhönen et al., 2019). Interestingly, increased BDNF levels are also observed following numerous exercise paradigm interventions for CNS injuries and are directly linked to cellular, synaptic, and anatomical plasticity as well as functional recovery after injury (Vaynman and Gomez-Pinilla, 2005; Houle and Côté, 2013). Upregulated BDNF is also observed in other rehabilitative strategies, including epidural spinal cord stimulation and cortical stimulation, which are used to promote motor recovery and pain control following SCI (Lynskey et al., 2008) or stroke (Bao et al., 2020). These findings, therefore, indicate that such rehabilitative strategies exert an immunomodulatory effect at the neurotrophic-neuroimmune axis and modify plasticity in spinal and cortical circuits.

Although exercise is an appealing, non-invasive rehabilitative strategy, there is a pressing need to delineate the therapeutic window for exercise initiation and how different forms of exercise (e.g., aerobic vs. anaerobic) influence functional recovery and plasticity following CNS injury. Delayed initiation of exercise beginning 4–5 weeks post-injury is associated with reduced microglial activation and IL-1β levels (Piao et al., 2013), as well as increased expression of neurotrophic factors and recovery of motoneuron reflex activity (Houle and Côté, 2013). Conversely, delayed exercise is ineffective for reversing neuropathic pain development and associated nociceptive afferents plasticity (Detloff et al., 2016). Moreover, training initiated at chronic time points post-injury is associated with diminished efficacy and reduced functional recovery (Biernaskie et al., 2004; Norrie et al., 2005). Efforts seeking to expand this interventional window have highlighted the therapeutic potential of stimulating neuroinflammation to enhance plasticity. In a study by Fouad and colleagues (Torres-Espín et al., 2018), inflammation induced *via* systemic injection of lipopolysaccharide (LPS) improved the efficacy of rehabilitative training when initiated 8-weeks post-SCI. This was further correlated with increased sprouting of corticospinal axons and increased EMG activity following cortical stimulation. Thus, inflammation-induced plasticity may be utilized to enhance rehabilitative strategies in chronically injured individuals.

As emerging rehabilitative strategies continue to be developed for functional recovery of locomotion (e.g., powered exoskeletons), respiration (e.g., intermittent hypoxia), and modulation of spared neural tissue (e.g., nerve/cell grafts), these strategies will hopefully provide a deeper understanding of the mechanisms driving plasticity throughout the neural axis and how this shapes neural circuit activity. Furthermore, current and future research must also assess whether these therapeutic strategies also drive maladaptive plasticity and detrimental outcomes, as many targets for adaptive plasticity can induce unwanted effects (e.g., intraspinal axonal sprouting to promote motor recovery may also exacerbate sympathetic hyperreflexia and episodes of autonomic dysreflexia).

## Conclusions

Activation of the neuroimmune system following an injury to the CNS drives widespread plasticity throughout the neuraxis to ultimately modulate neural circuit activity and functional activity. Through direct and indirect mechanisms, this plasticity results in altered homeostasis of presynaptic and postsynaptic cells which contribute to overall increased excitability and diminished inhibition at a cellular and circuit-wide level. This heightened cell excitability and activation further drive synaptic remodeling and fiber sprouting, which leads to the formation of new synapses, shifts in fiber phenotypes, and reorganization of cortical, subcortical, and intraspinal anatomy. Such cellular, synaptic, structural, and anatomical plasticity feeds into multiple neural circuits to ultimately induce widespread physiological outcomes that can be adaptive and/or detrimental depending on the context. As advancements in the neurotrauma field continue to elucidate the mechanisms driving adaptive and maladaptive plasticity, further research is needed to understand how the neuroimmune system affects specific neuronal subtypes to alter circuit-wide activity, and how the severity, level, and location of different injury types may alter this plasticity. Furthermore, although researchers are often cognizant of the dichotomous implications of injury-induced plasticity, therapeutic strategies for the treatment of maladaptive functional outcomes (e.g., neuropathic pain, spasticity, hyperreflexia, autonomic dysreflexia) or promotion of adaptive functional activity (e.g., recovery of locomotion, micturition, and respiration) must be carefully and thoroughly explored to avoid or minimize potential contraindications. Research that further investigates the mechanistic and therapeutic modalities of the neuroimmune system will provide enormous value and insight into the complex mechanisms attributed to circuit plasticity and spontaneous recovery of function after CNS injury.

## Author Contributions

MO’R and VT wrote the manuscript.

## Conflict of Interest

The authors declare that the research was conducted in the absence of any commercial or financial relationships that could be construed as a potential conflict of interest.

## Abbreviations

CNS: central nervous system
TBI: traumatic brain injury
SCI: spinal cord injury
BBB: blood-brain barrier
BSB: blood-spinal barrier
BCSF: blood-cerebrospinal fluid barrier
CCLx: chemokine ligand family
CXCLx: chemokine (C-X-C motif) ligand family
IL-x: interleukin family
TNFα: tumor necrosis factor-alpha
IFNγ: interferon-gamma
NF-κB: nuclear factor kappa-light-chain-enhancer of activated B cells
TGF-β1: transforming growth factor-beta 1
Tmem119: transmembrane protein 119
ROS: reactive oxygen species
BDNF: brain-derived neurotrophic factor
GDNF: glial cell-line derived neurotrophic factor
NGF: nerve growth factor
NT-3: neurotrophin-3
MMP-9: matrix metalloproteinase-9
Trk: tropomyosin-related kinase
CSPG: chondroitin sulfate proteoglycans
EAAT: excitatory amino acid transporter
EPSC: excitatory post-synaptic current
EPSP: excitatory post-synaptic potential
IPSP: inhibitory post-synaptic potential
GABA: γ-aminobutyric acid
VGAT: vesicular GABA transporters
KCC2: potassium-chloride cotransporter
TSP: thrombospondin
CGRP: calcitonin gene-related peptide
TRPV1: transient receptor potential cation channel subfamily V member 1
VIP: vasoactive intestinal peptide
IB4: isolectin B4
PACAP: pituitary adenylate cyclase-activating polypeptide
ACC: anterior cingulate cortex
S1: primary somatosensory cortex
SSR: spinal sympathetic reflex
EMG: electromyography.
